# Mr. Morrison's Case of Tinea Capitis

**Published:** 1809-03

**Authors:** Thomas Morrison

**Affiliations:** Dublin


### Medical and Physical Journal.

VOL. XXI.] MARCH, 1809. [NO. 121.

*Printed for R. PHILLIPS, by W. Thorne, Red Lion Court, Fleet Street, London.*

### *To the Editors of the Medical and Physical Journal.*

GENTLEMEN,

THE friends of B. D. aged about 16, made application to me concerning his disease, which was a case of inveterate Tinea Capitis, attended with those troublesome symptoms observable on such occasions; such as extreme itching, heat, &c. On a minute inquiry into the progress of this formidable complaint, I was told, that he had applied to every medical and surgical advice that this great metropolis could afford, without the necessary relief, and that it had continued uninterruptedly for about four years.

When I carefully examined the parts affected, I perceived the entire scalp to be overspread with a loathsome incrustated appearance, and that it emitted at the same time a singularly offensive fœtor; there was, morever, a disposition to debility in his habit of body; the digestive powers were much impaired, and many predisposing marks of a scrofulous constitution were apparent.

Though an intimate acquaintance with the structure of the human body is indispensably necessary to qualify a man for becoming a good surgeon; yet many who are not adepts in anatomy, may be instructed so as to assist their fellow creatures in cases of emergency. The operation for perfecting a radical cure in cases of tinea capitis, I have seen performed by persons unacquainted with the profession, and with amazing facility.

I gave directions to the subject of this case, to have his head as closely shaved as was possible, and in such parts thereof as the razor could not touch, the scissars was necessarily substituted; a common poultice was then applied over the parts affected, at the same time my patient was advised to call upon me in the course of a few days.

When he came as instructed, I observed that the poultice had effected its purpose, so far as to render soft the incrustation, yet it exhibited a very foul appearance, and with little or no diminution of fœtor; also, the scabs continued to rise higher and thicker above the surface.

After another careful removal of the hair, and the head being well washed with warm soap and water, I applied the paste compounded as below, and spread on strips of strong linen. Of yellow resin, two ounces; of best ale, one pound; of the finest flour, three ounces. To the melted resin, add the ale and flour gradually, the two latter ingredients having been previously intermingled in a bason together.

Each morning I removed the paste, strip after strip, which gave, on the first applications, some degree of pain and uneasiness. It was also attended with a slight effusion of blood; yet he told me with much satisfaction after its removal, that his head was much easier than he had remembered it since the commencement of the disease.

I cautiously and attentively, for three weeks, removed and re-applied this adhesive paste, observing that my patient was less affected with pain after each succeeding application; I also, with a pair of scissars, clipped off the hair which began to grow, and gently separated such rising parts as might prevent the adhesion of the paste. From the first application to that period wherein I could pronounce a perfect cure, the effusion of blood was observed to diminish gradually, that had issued on the first dressings, and all other appearances proceeded favourably.

Some years ago, I inserted a paper in the Annals of Medicine of Edinburgh, on the foregoing disease, which has since been transcribed into the fourth edition of Doctor Underwood's Treatise on the Diseases of Children. In that case, with the account therein given of its radical cure by the above paste, I perceive, what must have been *an error of the press,* the yellow resin is directed to be added to the other ingredients; the intention (according to the rules of pharmacy) was to have the *resin* first dissolved, and to add the thinnest part of the ale and flour gradually; continually stirring it in a brass skillet, on a brisk fire, until the whole be perfectly incorporated, and assume a thick gelatinous appearance. The paste was directed to be spread as above and renewed each day, whilst at the same time, the head was to be rubbed well with a coarse cloth, towards the termination of the disease. By this mode of procedure, procedure, I have radically cured, in about eleven years, forty-eight patients; and, as far as I can understand, not one of them has had the least return of the complaint; they most generally enjoy good health, and have remarkably fine hair.

I am, &c.

THOMAS MORRISON.

*Dublin, January* 3, 1809.

